# Amino Acids as Multifunctional Molecules in Plants: From Fundamental Metabolism to Precision Agriculture

**DOI:** 10.3390/plants15101583

**Published:** 2026-05-21

**Authors:** Zhaofeng Wang

**Affiliations:** Guangdong Provincial Key Laboratory of Plant Molecular Breeding, College of Agriculture, South China Agricultural University, Guangzhou 510642, China; wangzhaofeng@scau.edu.cn

**Keywords:** amino acids, biological functions, signal transduction, stress response

## Abstract

Amino acids are organic compounds that serve as the fundamental building blocks of proteins and are additionally responsible for a multitude of other biological functions. This review synthesizes recent evidence elucidating that amino acids function as vital players in nitrogen transport, stress defense, and perhaps most intriguingly as signaling molecules. For example, glutamate triggers calcium signals through GLR receptors to guide root growth and pollen tubes. Others, like proline and glutathione, protect cells from drought, salt, and oxidative damage. Aromatic and sulfur-containing amino acids also feed into the production of hormones (auxin, ethylene) and a wide range of defense compounds. Beyond metabolism, we highlighted how plants sense amino acid status via ancient sensors such as PII and the TOR pathway, which fine-tune growth and resource allocation. Understanding this hidden side of amino acids opens new doors for agriculture. We discussed how these insights could lead to smarter biostimulants, gene-edited crops with better nutrient efficiency, and nano-based delivery systems. In short, amino acids are not just food for plants—they are signals, shields, and switches that shape how plants grow and cope with stress.

## 1. Introduction

Amino acids are universally recognized as the building blocks of proteins, a role that has dominated plant physiology and biochemistry for decades. Through peptide bond formation, the twenty standard amino acids polymerize into polypeptide chains that fold into complex three-dimensional structures, enabling enzymatic catalysis, cellular architecture, and signal transduction [1,2]. Yet, confining amino acids to this single function—essential as it is—oversimplifies their biological repertoire. Over the past two decades, growing evidence has revealed that amino acids in plants also participate in nitrogen transport, stress protection, hormone synthesis, secondary metabolism, and direct signaling. This has shifted the view of amino acids from passive substrates to active regulators that coordinate growth, development, and environmental responses.

A key reason for this multifunctionality lies at the intersection of plant carbon and nitrogen metabolism. Plants absorb inorganic nitrogen (nitrate and ammonium) through their roots and assimilate it primarily into glutamate and glutamine via the glutamine synthetase (GS)/glutamate synthase (GOGAT) cycle [3]. From this hub, nitrogen is channeled into all other amino acids and then into proteins, nucleic acids, and countless nitrogen-containing metabolites. Consequently, amino acids—particularly glutamine (Gln) and asparagine (Asn)—serve as the major forms for long-distance nitrogen transport between source and sink organs and for nitrogen storage in seeds [4]. This central position in primary metabolism means that amino acid pools reflect the plant’s overall carbon-nitrogen balance, and any fluctuation in their levels can act as a metabolic signal. In essence, the very architecture of plant nitrogen metabolism predisposes amino acids to function as information carriers.

The signaling roles of amino acids are now being uncovered at multiple levels. Some act as direct precursors for phytohormones—tryptophan for auxin, methionine for ethylene, arginine for polyamines—linking primary metabolism to developmental programs [5]. Others, such as glutamate, function as extracellular signals through dedicated receptor-like channels (the GLR family), triggering rapid Ca^2+^ fluxes that regulate root architecture, pollen tube guidance, and defense responses [6,7]. Inside the chloroplast, the ancient PII protein senses glutamine levels and adjusts arginine biosynthesis and fatty acid metabolism accordingly [8]. At the whole-plant level, the Target of Rapamycin (TOR) kinase integrates amino acid availability with energy status to orchestrate growth, translation, and autophagy [9]. It is striking that these diverse signaling mechanisms operate on vastly different timescales—from milliseconds (GLRs) to minutes (PII) to hours or days (TOR)—suggesting that plants use amino acids as both rapid emergency signals and long-term nutritional gauges.

Beyond metabolism and signaling, amino acids are indispensable for plant survival under stress. Under drought, salinity, or cold, compatible solutes such as proline accumulate to high levels, stabilizing proteins and membranes while scavenging reactive oxygen species [10]. Glutathione, a tripeptide derived from glutamate, cysteine, and glycine, is the cell’s primary redox buffer and is essential for detoxifying heavy metals and pathogens [11]. Non-protein amino acids like canavanine act as anti-herbivore toxins, while amino-acid-derived secondary metabolites—glucosinolates, alkaloids, phytoalexins—form the chemical backbone of plant immunity [12]. That a single class of molecules can function as structural components, transport vehicles, signals, and defense compounds is remarkable, yet it raises a fundamental question: how do plants balance these competing demands without depleting the amino acid pools needed for protein synthesis? This question has received surprisingly little attention, and most studies still examine one function in isolation.

This review synthesizes recent advances that collectively argue for a paradigm shift: amino acids should be viewed not merely as protein building blocks but as multidimensional regulatory hubs that integrate carbon-nitrogen status, stress adaptation, and developmental decisions. We begin by revisiting the core nutritional functions of amino acids in nitrogen assimilation, transport, and protein synthesis, with an emphasis on metabolic flexibility and the recent discovery of aminotransferase substrate promiscuity. We then discuss their roles as precursors of bioactive molecules, including hormones and secondary metabolites, highlighting where pathway regulation differs between model and crop species. A major focus is placed on emerging signaling functions mediated by GLRs, PII, and TOR, where we critically assess the evidence for functional specificity and remaining gaps. We then survey the protective roles of amino acids in abiotic and biotic stress responses, emphasizing often overlooked trade-offs and metabolic costs. Finally, we discuss current challenges, including the predominance of *Arabidopsis*-centric studies, the lack of systems-level integration, and the need to translate mechanistic insights into agricultural applications such as precision biostimulants and gene editing. Throughout, we highlight unresolved questions and propose future research directions that go beyond cataloging functions toward understanding how plants orchestrate the competing demands placed on their amino acid pools.

## 2. Multifunctional Roles of Amino Acids in Plant Metabolism, Signaling, and Stress Responses

### 2.1. The Core Functions of Amino Acids: Protein Synthesis and Nitrogen Metabolism

The most familiar function of amino acids is also the most fundamental: they are the substrates for protein synthesis. Every polypeptide chain is assembled from the twenty standard amino acids through a highly conserved, energy-intensive process. Aminoacyl-tRNA synthetases first activate each amino acid by linking it to its cognate tRNA, consuming ATP [13]. The ribosome then reads the messenger RNA triplet by triplet, adding amino acids to the growing chain according to the genetic code [14]. Peptide bond formation is catalyzed by the ribosome’s peptidyl transferase activity, with the chain elongating through cycles of aminoacyl-tRNA binding, peptide transfer, and translocation [15]. Termination occurs when a stop codon is reached, and release factors hydrolyze the completed polypeptide from the tRNA [16]. This machinery is so deeply embedded in plant biology that it is easy to take for granted. Yet, the sheer demand for amino acids to sustain protein synthesis imposes a constant pressure on plant nitrogen metabolism. Under nitrogen limitation, translation is one of the first processes to slow, and seed storage proteins become a critical reserve for early seedling growth [17]. In this sense, protein synthesis is not merely a consumer of amino acids but also a regulator that couples plant growth to nitrogen availability. A theme running through this entire review is how plants secure the amino acid supply for translation and prioritize it over other uses such as secondary metabolism or signaling.

**Figure 1:**
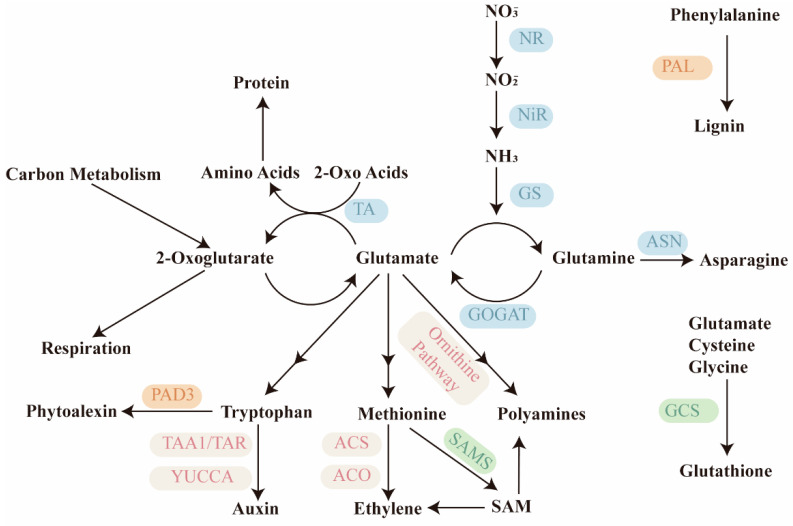
**Integration of nitrogen assimilation, amino acid metabolism, and downstream secondary pathways in plants.** Schematic representation of primary and secondary metabolism centered on glutamate (Glu) and glutamine (Gln) in plants. Inorganic nitrate (NO_3_^−^) is reduced to nitrite (NO_2_^−^) and then to ammonium (NH_3_) by nitrate reductase (NR) and nitrite reductase (NiR). Ammonium is assimilated into Gln via glutamine synthetase (GS); together with 2-oxoglutarate, Gln is converted by glutamate synthase (GOGAT) into two molecules of Glu, forming the GS/GOGAT cycle that serves as the entry point of reduced nitrogen into organic metabolism. Glu and Gln act as central hubs connecting carbon skeletons (2-oxoglutarate) to the biosynthesis of all proteinogenic amino acids, including proline, ornithine/arginine, methionine, tryptophan, and asparagine (via ASN). From these, multiple bioactive molecules branch off: tryptophan is converted to auxin (via TAA1/TAR and YUCCA) and the phytoalexin camalexin (via PAD3); methionine gives rise to S-adenosylmethionine, which feeds into ethylene (ACS, ACO) and polyamine synthesis; glutamate, cysteine, and glycine combine to form glutathione (γ-glutamylcysteine synthetase, GCS); and phenylalanine is directed to lignin via phenylalanine ammonia-lyase (PAL). The diagram thus illustrates how the GS/GOGAT cycle coordinates nitrogen and carbon status to regulate growth, development, and stress defense through interconnected primary and specialized metabolic routes. TA, transaminase; SAMS, SAM synthetase.

The central position of amino acids in nitrogen metabolism explains their tight link to overall plant performance. Plants take up inorganic nitrogen—mostly nitrate and ammonium—from the soil and convert it into organic form through the GS/GOGAT cycle. Ammonium is first incorporated into glutamate to yield glutamine via GS, after which GOGAT transfers the amide group to α-ketoglutarate, producing two molecules of glutamate (Figure 1) [18]. This cycle is the gateway through which almost all nitrogen enters plant metabolism. From glutamate and glutamine, nitrogen is distributed to other amino acids through transamination reactions, a process dependent on carbon skeletons from photosynthesis and respiratory pathways (Figure 1) [19]. Because the GS/GOGAT cycle operates in both roots and shoots, it ties whole-plant nitrogen assimilation to the production of transport-competent amino acids. Indeed, glutamine and asparagine—the latter synthesized by asparagine synthetase (ASN)—serve as the primary long-distance nitrogen carriers in the xylem and phloem (Figure 1) [4]. Their high nitrogen-to-carbon ratio, good solubility, and chemical stability make them ideal for moving nitrogen from source tissues to sink tissues [20]. When nitrogen fertilizer is applied, the expression of ASN and GS genes is rapidly upregulated, driving asparagine and glutamine accumulation and promoting grain nitrogen content [20]. Conversely, under nitrogen deficiency, these amino acids are remobilized from senescing leaves, underscoring their role as mobile nitrogen stores. However, the efficiency of this remobilization varies greatly among species and even cultivars, a fact that has received surprisingly little attention in breeding nitrogen-efficient crops.

It is at the intersection of carbon and nitrogen metabolism that amino acids reveal their most integrative role. All twenty standard amino acids derive their carbon skeletons from intermediates of glycolysis, the tricarboxylic acid (TCA) cycle, and the pentose phosphate pathway [19]. The synthesis of any given amino acid therefore requires not only sufficient nitrogen but also an adequate flow of carbon through central metabolism. Conversely, when amino acids accumulate to high levels, they feedback-inhibit nitrate uptake and nitrate reductase (NR) activity, preventing the plant from assimilating more nitrogen than its carbon skeletons can accommodate [21]. This homeostatic mechanism ensures a balanced allocation of carbon and nitrogen resources, but it also creates a potential bottleneck: under low light, nitrogen assimilation slows even if nitrogen is abundant. What is less appreciated is that the robustness of this metabolic network may depend on a recently characterized property of aminotransferases. Koper and colleagues mapped the substrate specificity of *Arabidopsis* aminotransferases and discovered that many exhibit broad substrate promiscuity, catalyzing transamination reactions with multiple different amino acid pairs. This promiscuity enhances the redundancy and flexibility of the nitrogen metabolic network, allowing plants to cope with fluctuating nitrogen sources and carbon availability more effectively than if each enzyme were highly specialized [22]. The implication is that the network’s resilience is an emergent property of enzyme substrate ambiguity, challenging the conventional view of metabolic pathways as rigid, linear sequences.

The practical implications of understanding these core functions are most evident in efforts to improve nitrogen use efficiency (NUE) in agriculture. Applying amino-acid-based biostimulants has been shown to enhance NUE by activating key enzymes such as nitrate reductase and glutamine synthetase, promoting photosynthesis and biomass accumulation, and reducing nitrate accumulation in edible tissues [23]. These effects are often attributed to amino acids serving as direct nitrogen sources, but that interpretation is too simplistic. Exogenously applied amino acids can also act as signals that reprogram plant metabolism, inducing the expression of transporter and assimilation genes. Distinguishing between the nutritional and signaling effects of applied amino acids is a challenge the field is only beginning to address. Moreover, the substrate promiscuity of aminotransferases suggests that metabolic engineering efforts aimed at increasing a single amino acid may have unintended consequences on the pools of other amino acids [24]. A deeper, network-level understanding of amino acid metabolism will be required to design crops with both high NUE and high nutritional quality.

In summary, amino acids are not merely the building blocks of proteins but are also central integrators of plant carbon and nitrogen metabolism, with their synthesis, transport, and recycling governed by a flexible enzyme network that underpins nitrogen use efficiency and offers targets for metabolic engineering.

### 2.2. Amino Acids as Precursors of Bioactive Molecules

If the core functions in protein synthesis and nitrogen transport represent the “housekeeping” roles of amino acids, then their capacity to serve as precursors for bioactive molecules is where multifunctionality truly expands. A handful of amino acids are channeled into essential phytohormones: tryptophan to auxin [5,25], methionine to ethylene [26,27], arginine and glutamate to polyamines [28,29]. Others feed into vast networks of secondary metabolism, giving rise to lignin [30,31], flavonoids [32], glucosinolates [33,34], alkaloids [35,36], and phytoalexins [37,38]—compounds that shape plant architecture, defense, and environmental interactions. Still others are converted into small but indispensable molecules such as S-adenosylmethionine (SAM), the universal methyl donor, and glutathione (GSH), the central redox buffer [11,39]. At first glance, these pathways appear as a collection of independent chemical conversions.

Three deeper insights emerge when these precursor pathways are examined together. First, the same amino acid often feeds into multiple, sometimes competing, fates. Methionine, for instance, is the starting point for ethylene, polyamines (via SAM), and glucosinolates [26,28,33]. Tryptophan contributes to auxin, indole glucosinolates, and the phytoalexin camalexin [5,25,38,40]. The regulatory mechanisms that direct flux toward one product or another likely involve a combination of transcriptional control, substrate channeling, and post-translational regulation of branch-point enzymes [41,42,43]. Second, the genetic and biochemical logic of these pathways differs substantially between model systems and crops, and even among crop species. The phenylpropanoid pathway is broadly conserved, but the relative importance of lignin versus anthocyanin synthesis varies with species, tissue, and stress history [30,31]. More dramatically, the major rice phytoalexins are diterpenoids (momilactones), not tryptophan-derived compounds like camalexin in *Arabidopsis*, indicating that the chemical arsenal of plant immunity is lineage-specific [4,38]. Generalizing from *Arabidopsis* to all flowering plants can therefore be misleading. Third, the boundary between “primary” and “secondary” metabolism is more porous than traditionally taught. The same precursor amino acid participates in both, and the enzymes that commit an amino acid to a secondary pathway often evolved from primary metabolic enzymes through gene duplication and neofunctionalization [30,43]. Understanding how plants balance amino acid allocation between growth and defense is a central question in plant biology with direct implications for crop improvement. Trade-offs are inevitable: a plant investing heavily in lignin or glucosinolates has fewer amino acids available for protein synthesis. Yet, the molecular mechanisms mediating such trade-offs remain largely unexplored, with most studies focusing on one pathway at a time [23,44]. The following subsections illustrate these principles through specific examples.

#### 2.2.1. Hormone Synthesis Precursors

Several essential phytohormones originate directly from specific proteinogenic amino acids, yet the regulatory logic governing their production differs fundamentally among pathways. Auxin is synthesized primarily from tryptophan via the indole-3-pyruvic acid (IPA) pathway, catalyzed sequentially by TAA1/TAR aminotransferases and YUCCA flavin monooxygenases (Figure 1) [5,25]. This pathway operates under tight local and tissue-specific control: transcriptional regulation, post-translational modification, and feedback inhibition by IAA collectively generate auxin maxima that direct organ initiation, tropic responses, and vascular patterning [45,46]. 

However, a persistent question is whether local biosynthesis alone accounts for the spatial precision of auxin distribution, or whether tryptophan-independent routes contribute under specific conditions such as wounding or pathogen infection [4]. Moving from auxin to ethylene, the regulatory strategy shifts from localized biosynthesis to post-translational control of enzyme stability. Ethylene is produced from methionine via SAM, with ACC synthase (ACS) and ACC oxidase (ACO) as the dedicated enzymes (Figure 1) [26,27]. Unlike the transcriptional dominance in auxin synthesis, ethylene production is primarily gated by the rapid turnover of ACS proteins through the ubiquitin-proteasome pathway [47,48]. This mechanism enables plants to mount fast, reversible ethylene bursts in response to mechanical stress, flooding, or pathogen attack [1]. Nevertheless, recent studies in rice have shown that transcriptional regulation of *OsACS* and *OsACO* also plays a significant role under specific stresses, indicating that the balance between transcriptional and post-translational control is species- and context-dependent [16]. Furthermore, cytokinins modulate both ACS and ACO activity in a spatially discrete manner along the root axis, implying that ethylene biosynthesis is integrated into a broader hormonal network [49].

A third, often overlooked, dimension involves polyamines—putrescine, spermidine, and spermine—which are synthesized from arginine (via ADC) or glutamate (via the ornithine pathway) (Figure 1) [28,29]. Unlike auxin and ethylene, which act at sub-micromolar concentrations, polyamines accumulate to millimolar levels and function as cationic molecules that stabilize nucleic acids and membranes [50]. However, a critical twist is that polyamine catabolism generates hydrogen peroxide (H_2_O_2_), positioning polyamines at the crossroads of growth regulation and oxidative signaling [2]. Moreover, polyamines and ethylene share SAM as a common precursor, creating a competitive metabolic relationship: enhanced ethylene production often suppresses polyamine accumulation, and vice versa (Figure 1) [39]. This trade-off has profound implications for stress adaptation—for instance, during pathogen attack, the shift toward ethylene-mediated defense may come at the expense of polyamine-dependent membrane protection, a cost only beginning to be quantified.

Comparing these three pathways reveals not a simple list of precursor-to-product conversions, but a dynamic, interconnected network where different regulatory logics coexist and compete. Unresolved questions abound. First, how do these pathways partition shared carbon and nitrogen resources under fluctuating environmental conditions? Second, what molecular mechanisms integrate auxin, ethylene, and polyamine signaling into coherent developmental outputs? Recent studies hint at such integration: ethylene promotes auxin biosynthesis by upregulating the tryptophan biosynthetic gene *ASA1* through the transcription factor ERF1, thereby linking ethylene signaling to auxin production [51]. Similarly, the *wei* mutants in *Arabidopsis* have revealed extensive feedback loops between ethylene and auxin [51]. Yet, a systems-level understanding of how plants coordinate these competing demands—and how such coordination differs between *Arabidopsis* and major crops such as rice, maize, and tomato—remains a priority for future research [52].

#### 2.2.2. Secondary Metabolite Synthesis Precursors

Unlike hormone pathways where a single amino acid typically feeds into a single signaling molecule, secondary metabolite biosynthesis is characterized by branching pathways that generate enormous chemical diversity from a limited set of precursors. Two interconnected questions lie at the heart of this field: how do plants control flux distribution among competing branches, and to what extent are these regulatory mechanisms conserved across species?

The phenylpropanoid pathway offers a striking example of branching logic. Phenylalanine and tyrosine serve as entry points into a vast network producing lignin, flavonoids, anthocyanins, and a wide range of phenolic compounds [30,31]. The first committed enzyme, phenylalanine ammonia-lyase (PAL), has long been considered the rate-limiting step (Figure 1). However, recent genomic analyses have complicated this view. In Epimedium pubescens, seven PAL genes have been identified with distinct evolutionary origins and expression patterns: two ancestral copies evolved under strong purifying selection, while others arose through segmental and tandem duplications [53]. This suggests that PAL family expansion—rather than the activity of a single enzyme—may provide the regulatory flexibility needed to partition carbon flux into different branches. Yet a critical question remains: what determines whether phenylalanine is channeled into lignin, anthocyanins, or salicylic acid? Recent evidence points to MYB transcription factors as key regulators, but the mechanisms integrating developmental, hormonal, and environmental signals at these branch points are only beginning to be understood [54].

Moving to methionine- and tryptophan-derived glucosinolates, the regulatory logic shifts from transcriptional control of a gateway enzyme to substrate specificity at the entry level. Glucosinolates are sulfur-containing defense co phytoalexins (momilactones) in rice mpounds characteristic of the *Brassicales* order, synthesized from methionine, tryptophan, or phenylalanine [33,34]. The entry point is determined by CYP79 family cytochromes P450, each of which accepts a specific amino acid as substrate (Figure 1) [55]. Unlike PAL, which converts a single substrate into a common intermediate that then diverges, the glucosinolate pathway uses different CYP79 isoforms to direct flux from different amino acids into the same core structure. Phylogenomic studies have revealed that small-scale duplications played a prominent role in the evolution of this pathway, and that gene loss contributed significantly to metabolic diversity across Brassicales genera [55]. In cabbage, exogenous hormone treatments (MeJA, ethylene) differentially regulate side-chain extension genes versus core structure genes, indicating that multiple regulatory layers operate in parallel [50]. The key open question is whether this pathway is a fixed endpoint or a launching pad for creating entirely new metabolites [55].

A third dimension comes from alkaloids, which incorporate nitrogen from arginine or ornithine into complex heterocyclic structures with potent biological activities [35,36]. In tobacco, the defensive alkaloid nicotine derives its pyrrolidine ring from putrescine, which can be synthesized from arginine (via ADC) or ornithine (via ODC) [56]. Classic biochemical studies suggested that the ADC route is preferentially used for nicotine production, but antisense suppression of ADC in tobacco hairy roots revealed only minor reductions in nicotine levels, while ODC suppression had much stronger effects [36,56]. This finding challenges the assumption of pathway specialization and raises a more general question: to what extent do ADC and ODC represent functionally redundant routes versus tissue- or condition-specific contributions? In animals and microbes, only one pathway is present, whereas plants retain both—suggesting that maintaining two parallel routes to putrescine may confer metabolic robustness under fluctuating nitrogen availability. However, direct comparative studies across species are lacking.

Comparing these three systems—phenylpropanoids, glucosinolates, and alkaloids—reveals shared principles and fundamental differences. All three must solve the same problem: how to take a common amino acid precursor and generate diverse end products without depleting the pool needed for primary metabolism. Yet the solutions differ. Phenylpropanoid diversity arises from transcriptional regulation of a single-entry enzyme (PAL) followed by branch-specific decoration. Glucosinolate diversity relies on substrate-specific CYP79 isoforms channeling different amino acids into a common core. Alkaloid diversity is constrained by the dual-route biosynthesis of putrescine, whose relative contribution varies by species and tissue. Unresolved questions remain. First, how do plants balance the carbon and nitrogen demands of secondary metabolism against those of growth and reproduction? Recent studies show that defense induction often represses primary metabolic genes, but the regulatory nodes mediating this trade-off are still being identified [23,44]. Second, are the regulatory principles derived from *Arabidopsis* applicable to crops? In rice, the major phytoalexins are diterpenoids rather than tryptophan-derived compounds, indicating that while the general strategy of amino-acid-derived defense is conserved, the specific end products and their regulatory logics may differ substantially between monocots and dicots [57]. Addressing these questions will require comparative studies across diverse plant lineages, combined with systems-level approaches tracking metabolic flux in real time.

#### 2.2.3. Other Important Molecular Precursors

Beyond their roles as hormone precursors and building blocks for secondary metabolites, certain amino acids serve as the source of small, highly versatile molecules that participate in virtually every aspect of cellular biochemistry. Two such molecules—S-adenosylmethionine (SAM) and glutathione (GSH)—deserve special attention because they illustrate how a single amino acid derivative can acquire multiple, seemingly unrelated functions through evolutionary tinkering.

SAM is synthesized from methionine and ATP in a reaction catalyzed by SAM synthetase (Figure 1) [39]. It is best known as the universal methyl donor for transmethylation reactions affecting DNA, RNA, proteins, lipids, and secondary metabolites. This function alone makes SAM indispensable for epigenetic regulation and metabolic control. However, SAM is also the direct precursor for ethylene (via ACS) and polyamines (via decarboxylated SAM), as discussed in Section 2.2.1. That a single molecule feeds into three distinct pathways—methylation, a gaseous hormone, and growth-regulating polyamines—creates a complex metabolic hub requiring careful regulation. A fundamental question is how plants partition SAM among these competing uses. Early studies suggested SAM levels are maintained within a narrow range, implying tight homeostatic control [39]. More recent flux analyses, however, have revealed that plants can rapidly shift SAM allocation in response to environmental cues: under pathogen attack, flux toward ethylene increases at the expense of polyamine synthesis, whereas under oxidative stress, flux toward glutathione is prioritized [2]. The regulatory mechanisms enabling this flexible partitioning—whether through allosteric control, substrate competition, or transcriptional reprogramming—remain largely unexplored. Moreover, SAM-dependent methylation consumes the methyl group but leaves the adenosyl backbone, which is recycled via the Yang cycle. Disruptions in this recycling pathway lead to the accumulation of toxic intermediates, and mutations in Yang cycle enzymes cause severe developmental phenotypes [39]. This recycling efficiency thus represents an additional, often overlooked, layer of regulation.

Glutathione (GSH) is a tripeptide (γ-Glu-Cys-Gly) synthesized from glutamate, cysteine, and glycine in two ATP-dependent steps catalyzed by γ-glutamylcysteine synthetase (GCS) and glutathione synthetase (GS) (Figure 1) [11,58]. Unlike SAM, which participates in multiple anabolic pathways, GSH functions primarily as a cellular redox buffer. The cysteine thiol group can be reversibly oxidized to glutathione disulfide (GSSG), and the ratio of GSH to GSSG serves as a key indicator of cellular redox status [11]. GSH is also the precursor of phytochelatins (PCs), which chelate heavy metals such as cadmium and arsenic [59]. The central importance of GSH in stress protection is underscored by the *Arabidopsis pad2* mutant, which carries a loss-of-function mutation in GCS1 and exhibits only 15–30% of wild-type GSH levels, leading to hypersensitivity to pathogens and heavy metals [59]. Yet, a critical question remains: how do plants coordinate GSH biosynthesis with the demand for its constituent amino acids, particularly cysteine? Cysteine is the product of a tightly regulated sulfur-assimilation pathway [60]. Thus, GSH levels are not controlled solely by GCS/GS activity but are indirectly constrained by sulfur nutrition and competition for cysteine between GSH and protein synthesis.

What connects SAM and GSH, despite their different functions, is the concept of metabolic competition for shared amino acid precursors. Both draw on the methionine-cysteine pool, albeit at different points. Therefore, enhanced demand for GSH—for instance, under oxidative stress—could theoretically limit SAM production, and vice versa. However, direct evidence for such a trade-off in plants is scarce. A notable exception is the finding that overexpression of GCS in *Arabidopsis* leads to increased GSH levels but reduced methionine and SAM levels, suggesting competition for cysteine is physiologically relevant [61]. Conversely, mutants with constitutive ethylene production often exhibit altered GSH pools [62]. These observations point to a hidden regulatory network linking sulfur assimilation, cysteine partitioning, and the balance between methylation capacity, ethylene signaling, and redox homeostasis. Unraveling this network—and determining how it differs between stress-tolerant and stress-sensitive species—represents an important direction for future research. Moreover, understanding how plants manage these molecules could inform metabolic engineering strategies for high-value production in plant-based systems [63].

In summary, the capacity of amino acids to serve as precursors for hormones, secondary metabolites, and small redox-active molecules illustrates a complex, interconnected network in which competing branch pathways, lineage-specific innovations, and trade-offs between growth and defense are governed by distinct regulatory logics that remain only partially understood.

### 2.3. Roles of Amino Acids in Signal Transduction

Amino acids function as highly specialized signaling molecules that enable plants to sense internal nutritional status and external environmental changes. Unlike the hormone precursors discussed in Section 2.2.1, the signaling roles of amino acids involve multiple, often overlapping, perception mechanisms operating at different spatial and temporal scales. Long-distance signals travel through the vasculature; local gradients guide developmental decisions at the tissue level; and intracellular sensors monitor metabolic flux. Unraveling how these distinct signaling layers are coordinated—and how they diverge between model and crop species—is a central challenge in plant signaling biology.

#### 2.3.1. As Long-Distance Transported Signaling Molecules

Plants transport amino acids between source and sink organs through the phloem and xylem to coordinate whole-plant nitrogen status. Glutamine (Gln) and asparagine (Asn), with their high nitrogen-to-carbon ratio, stability, and mobility, serve as the most important long-distance nitrogen carriers [4]. However, evidence has accumulated that these molecules do more than simply deliver nitrogen: their concentration ratios transmit information about nitrogen sufficiency or deficiency [64]. For example, the expression of asparagine synthetase (ASN) is regulated by carbonnitrogen balance, and the accumulation of Asn signals sink organs to adjust their metabolic programs under carbon-limited conditions [65].

A key unanswered question concerns the specificity of this long-distance signal. Do plants perceive the absolute concentration of a particular amino acid, the ratio of different amino acids (e.g., Gln/Glu), or the flux through a particular transporter? Studies using amino acid transporter mutants, such as *lht1*, have shown that local amino acid pools influence defense responses and systemic signaling [66], but whether these effects are due to altered transport per se or secondary changes in metabolism remains debated. Moreover, most of this work has been conducted in *Arabidopsis*, and it is unknown whether long-distance amino acid signaling in crops operates by similar principles. Comparative studies in rice, maize, and tomato are urgently needed.

#### 2.3.2. As Local Signals and Developmental Regulators

At the tissue and cellular levels, specific amino acids act as local instructive signals that pattern developmental processes with remarkable precision. Glutamate provides the most compelling example. A concentration gradient of glutamate exists in the pistil, and pollen tube tips express glutamate receptor-like (GLR) channels that perceive this gradient to guide growth toward the ovule [67,68]. This mechanism bears a striking analogy to axon guidance in the animal nervous system, yet it evolved independently in plants—a case of convergent evolution.

In the root system, glutamate signaling contributes to root architecture. AtGLR3.6 regulates root development and responsiveness to mechanical stimuli [69], while AtGLR3.4 and other GLRs participate in phloem-mediated signaling that affects lateral root initiation [6]. Disrupting GLR function alters root system architecture, demonstrating that local glutamate perception is functionally significant. What remains unclear is the relationship between these local signaling functions and the long-distance transport functions of the same amino acids. Are GLRs simply monitoring the concentration of glutamate arriving from distant tissues, or do they respond to locally synthesized pools that are functionally independent? Genetic approaches that separately manipulate local synthesis and long-distance transport will be required to dissect these possibilities.

#### 2.3.3. Receptors and Perception Mechanisms

The perception of amino acid signals in plants involves multiple receptor systems operating at the plasma membrane, within organelles, and as part of central signaling hubs. Three of the most intensively studied—the GLR family, the PII protein, and the TOR pathway—illustrate the hierarchical organization of amino acid signaling, from rapid ion fluxes to systemic growth regulation.

The plant GLR family consists of homologs of animal ionotropic glutamate receptors (iGluRs) and plays a vital role in plant cell signal transduction. The *Arabidopsis* genome encodes 20 *AtGLR* genes, phylogenetically classified into three clades based on sequence homology [70]. Clades I and III exhibit broad expression patterns across plant tissues, whereas Clade II expression is predominantly restricted to root tissues, suggesting potential functional specialization or redundancy [70,71]. Structurally, plant GLRs are integral membrane proteins of approximately 100 kDa, comprising several conserved domains: two extracellular ligand-binding domains (S1 and S2) forming a Venus flytrap motif for amino acid recognition, four transmembrane domains (M1–M4) with the M2 segment forming a re-entrant pore loop critical for ion permeation, and an N-terminal LIVBP-like domain proposed as a modulatory site for additional ligands [72,73]. Functional GLRs are believed to assemble as tetrameric complexes, analogous to animal iGluRs, facilitating combinatorial diversity in ligand sensitivity and channel properties [71,74].

**Figure 2:**
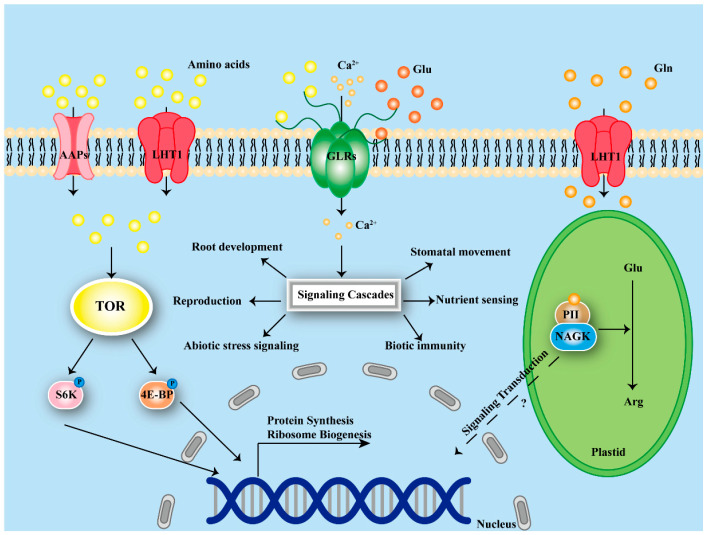
**Amino acid-mediated signaling pathways in plants.** The model summarizes key mechanisms by which amino acids function as signaling molecules. **Plasma Membrane Signaling:** Extracellular amino acids (e.g., glutamate) activate Glutamate Receptor-Like (GLR) ion channels, triggering Ca^2+^ influx and membrane depolarization that regulate processes such as pollen tube guidance, root architecture, and abiotic stress responses. **Intracellular Sensing:** The conserved PII protein in chloroplasts acts as a metabolic sensor, integrating signals of cellular energy (ATP/ADP) and carbon/nitrogen status (2-oxoglutarate, Glutamine) to regulate enzymes like *N*-acetylglutamate kinase (NAGK), thereby modulating arginine biosynthesis. **Systemic Growth Regulation:** Amino acid sufficiency promotes the activation of the Target of Rapamycin (TOR) kinase, a central regulator that stimulates anabolic processes (e.g., ribosome biogenesis, translation) and inhibits catabolic processes like autophagy, thereby coordinating growth with nutrient availability. S6K and 4E-BP, downstream target proteins of the TOR pathway; LHT1, lysine histidine transporter 1; AAPs, amnio acid permeases; Arg, arginine; Glu, glutamate; Gln, glutamine.

Unlike animal iGluRs, plant GLRs are not gated by classical agonists such as NMDA or AMPA [75]. Instead, they respond primarily to amino acids, including L-glutamate, glycine, serine, and asparagine [76,77]. Clade-specific variations in the ligand-binding domains underpin functional diversification; AtGLR1.4 exhibits a preference for hydrophobic amino acids like methionine, while AtGLR3.4 responds to serine and alanine [78,79]. This pharmacological diversity is further amplified by heteromeric assembly of GLR subunits. GLRs function as non-selective cation channels permeable to Ca^2+^, Na^+^, and K^+^ [80]. Electrophysiological studies confirm that GLR activation elicits rapid Ca^2+^ influx and membrane depolarization (Figure 2) [6,79]. A crucial unresolved question is how a single class of channels can mediate such diverse physiological processes. Recent evidence suggests functional specificity may arise from subcellular localization, assembly into distinct tetrameric complexes, or interaction with auxiliary subunits [81]. However, the molecular rules governing these interactions are only beginning to be understood.

The GLR family has been implicated in a wide range of processes—from root development and stomatal movements to reproduction, abiotic stress responses, and immunity (Figure 2). Part of the answer lies in their expression patterns: different GLRs are turned on in different cell types and at different developmental stages. For example, AtGLR3.3 and AtGLR3.4 are expressed in root tissues, where they regulate lateral root initiation through local Ca^2+^ signatures [6]. In contrast, AtGLR1.2 and AtGLR3.7 are active in pollen tubes, guiding growth toward the ovule [68]. Yet expression alone cannot explain everything. The same AtGLR3.3 that functions in roots also participates in pattern-triggered immunity in leaves, facilitating MAMP-induced Ca^2+^ influx and MAPK activation [82,83]. This suggests a single GLR can serve different purposes depending on cellular context. Adding to the complexity, GLRs are not confined to the plasma membrane. Alternative splicing of AtGLR3.5 generates isoforms targeted to mitochondria or chloroplasts, implying roles in organellar Ca^2+^ signaling that are only beginning to be explored [84]. Overexpression of rice *OsGLR1/2* enhances drought tolerance in both rice and *Arabidopsis*, indicating that at least some GLR functions are evolutionarily conserved [85]. At the same time, the diversity of GLRs across plant lineages—13 in rice, 20 in *Arabidopsis*, 34 in soybean—suggests lineage-specific expansions that may reflect adaptations to different ecological niches [86]. A critical unanswered question is whether these diverse functions arise from distinct GLR subunits operating in different cell types, or whether the same channels participate in multiple processes through different regulatory partners. Functional redundancy within the GLR family has made it difficult to assign specific functions to individual members using knockout mutants alone [78]. Moving forward, cell-type-specific rescue experiments and structural studies of different GLR tetramers will be needed to dissect the molecular basis of functional diversification.

While GLRs monitor extracellular amino acid concentrations, the PII protein functions as an intracellular metabolic sensor integrating carbon, nitrogen, and energy status within the chloroplast. PII is a highly conserved signaling molecule that orchestrates nitrogen metabolism across all domains of life [87,88]. In plants, PII has evolved to bind not only ATP/ADP and 2-oxoglutarate (2-OG)—a central metabolite reflecting carbon skeleton availability—but also glutamine, establishing it as a bona fide glutamine sensor [89,90]. A key mechanism underpinning PII’s role is its interaction with N-acetylglutamate kinase (NAGK), the rate-limiting enzyme in the ornithine/arginine biosynthesis pathway [91,92]. The formation of the PII-NAGK complex is enhanced by millimolar concentrations of glutamine, which bind to a specific C-terminal extension of the plant PII protein (Figure 2) [8]. Upon glutamine binding, the complex undergoes a conformational change that alleviates the feedback inhibition of NAGK by arginine, coupling high glutamine levels to upregulated arginine production. A notable exception to this conserved mechanism exists within the *Brassicaceae* family, including *Arabidopsis thaliana*, where the canonical glutamine-binding motif is not conserved [8]. This divergence raises a fundamental question: how does *Arabidopsis* sense glutamine status if its PII protein lacks the conserved binding site? Alternative pathways may operate but have not been identified, underscoring the danger of extrapolating from *Arabidopsis* to all plants. Beyond arginine biosynthesis, plant PII interacts with the biotin carboxyl carrier protein (BCCP) subunit of plastidial acetyl-CoA carboxylase (ACCase), thereby modulating de novo fatty acid biosynthesis [93]. Additional roles implicated in chloroplastic nitrite uptake and protein degradation further highlight PII’s role as a central metabolic hub [94,95].

The Target of Rapamycin (TOR) kinase operates at a different timescale and organizational level from GLRs and PII. While GLRs generate Ca^2+^ transients within milliseconds and PII adjusts metabolic flux over minutes to hours, TOR orchestrates the long-term decision of whether a plant should invest in growth or conserve resources. When amino acids are abundant, TOR is activated and promotes anabolic processes, phosphorylating downstream effectors such as S6K and 4E-BP to stimulate ribosome biogenesis and protein translation [96,97]. When amino acids become scarce, TOR activity drops, releasing the brake on autophagy—a recycling pathway that generates amino acids and energy (Figure 2) [98]. This growth-versus-survival switch is central to plant adaptation, yet the mechanism by which plants sense amino acid availability upstream of TOR remains mysterious. In animals and yeast, a well-characterized pathway involving Rag GTPases links amino acid levels to TOR. Plants possess homologs of some of these components, but whether they function analogously is not known [99]. Moreover, TOR integrates multiple nutrient signals simultaneously—not only amino acids but also sugars and nitrogen sources. When carbon is abundant but nitrogen islimiting, TOR activity is suppressed, indicating that plants weigh different inputs before committing to growth [100]. How this integration is achieved at the molecular level is an open question. Nearly all of our current understanding comes from *Arabidopsis* and tobacco cell cultures; it is unknown whether crop species have similar or divergent TOR signaling architectures. Natural variation in TOR pathway components could be a rich source of targets for breeding stress-resilient, high-yielding varieties.

Moving from individual receptors to an integrated signaling network, what emerges is a picture of temporal and spatial complementarity. GLRs provide rapid, local Ca^2+^ signals. The PII protein continuously monitors intracellular glutamine levels, adjusting metabolism on a slower timescale. TOR integrates these local and metabolic signals into systemic decisions about growth, autophagy, and resource allocation. Yet the connections between these layers are largely unexplored. Do GLR-generated Ca^2+^ signals feed into TOR activation? Does PII-mediated metabolic adjustment influence the availability of amino acids that activate TOR? Addressing these questions will require not only genetic dissection of each pathway in isolation but also systems-level approaches that track signaling flux across multiple scales simultaneously.

Thus, amino acid signaling in plants operates as a multi-temporal, multi-compartmental network: GLRs provide rapid local Ca^2+^ signals, PII monitors metabolic flux in chloroplasts, and TOR integrates these inputs to orchestrate long-term growth versus survival decisions—yet how these layers communicate remains a major open question.

### 2.4. Roles of Amino Acids in Response to Abiotic Stress

When plants encounter drought, high salinity, extreme temperatures, or heavy metal toxicity, they must mount a coordinated cellular defense. Among the many metabolic adjustments, changes in amino acid metabolism are among the fastest and most pronounced [101,102]. The same molecules that serve as protein building blocks and signaling agents are rapidly repurposed as osmoprotectants, antioxidants, metal chelators, and even alternative respiratory substrates. But this repurposing comes with costs: diverting amino acids into stress protection reduces their availability for growth. How plants weigh these competing demands is a central question in stress biology.

#### 2.4.1. Osmolytes: The Central Roles of Proline and Glycine Betaine

Under osmotic stress, plants accumulate compatible solutes to lower cellular osmotic potential, maintain turgor, and stabilize macromolecular structures. Two amino-acid-derived molecules, proline and glycine betaine, stand out for their quantitative importance and the depth of genetic evidence linking them to stress tolerance.

**Figure 3:**
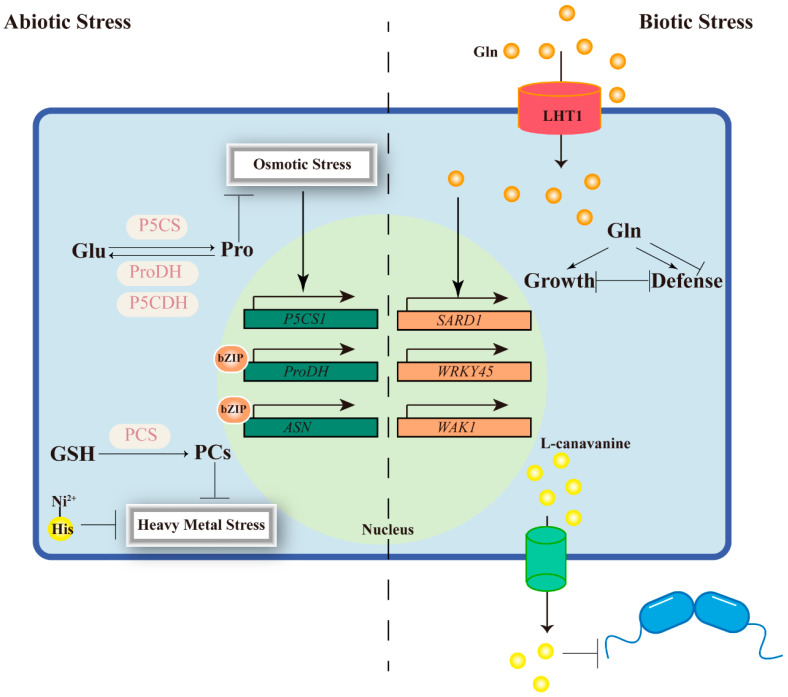
**Metabolic and transcriptional regulatory networks underlying plant responses to abiotic and biotic stresses.** Schematic model illustrating the major metabolic and transcriptional pathways that balance growth and stress tolerance in plant cells under abiotic (salinity, drought, heavy metals) and biotic (pathogens, insects) stress conditions. **Responses to abiotic stresses:** On the left side, osmotic stress directly upregulates P5CS1 to promote proline (Pro) synthesis from glutamate (Glu), while bZIP transcription factors regulate ProDH (involved in Pro degradation) and ASN (asparagine synthase). Under heavy metal stress, histidine (His) chelates nickel ions (Ni^2+^) to alleviate metal toxicity, and glutathione (GSH) is converted to phytochelatins (PCs) via PCS. **Responses to biotic stresses:** On the right side, the amino acid transporter LHT1 mediates glutamine (Gln) uptake; Gln acts as a signal that activates both growth-promoting and defense-related genes, including SARD1, WRKY45, and WAK1 in the nucleus. Meanwhile, the non-protein amino acid L-canavanine is imported and inhibits pathogen growth. The diagram highlights how plants integrate metabolic adjustments, amino acid signaling, and transcriptional reprogramming to cope with diverse environmental threats. P5CDH, pyrroline-5-carboxylate dehydrogenase; P5CS, Δ^1^-pyrroline-5-carboxylate synthetase; LHT1, lysine histidine transporter 1.

Proline accumulation is perhaps the best-characterized metabolic response to abiotic stress. The key enzyme is Δ^1^-pyrroline-5-carboxylate synthetase (P5CS), which catalyzes the rate-limiting step of proline biosynthesis (Figure 3). In *Arabidopsis*, *P5CS1* is strongly induced by drought, salinity, and abscisic acid (ABA), whereas *P5CS2* is more constitutive and essential for development under non-stressed conditions [10,103]. The physiological importance of *P5CS1* was demonstrated decades ago: antisense suppression leads to reduced proline accumulation, impaired osmotic adjustment, and enhanced wilting under drought (Figure 3) [104]. Conversely, removal of feedback inhibition on *P5CS* increases proline content and improves stress tolerance [105]. More recent work has shown that the *p5cs1* mutant is significantly more sensitive to PEG-mediated drought stress, and exogenous proline application restores only part of the wild-type tolerance, indicating proline acts through both osmotic and non-osmotic mechanisms [106].

Nearly as important as proline synthesis is its catabolism. Proline degradation occurs in the mitochondria via proline dehydrogenase (ProDH) and pyrroline-5-carboxylate dehydrogenase (P5CDH), generating glutamate that enters the TCA cycle (Figure 3). Under stress, *ProDH* expression is suppressed to allow proline accumulation; upon stress relief, *ProDH* is rapidly induced. The *prodh* loss-of-function mutant hyper-accumulates proline and shows enhanced drought and salt tolerance, but also exhibits developmental abnormalities and reduced seed set, illustrating the trade-off between stress protection and normal growth [107]. A less appreciated fact is that proline catabolism itself generates ROS in the mitochondria, and this must be managed by alternative oxidase (AOX) pathways. In *Arabidopsis*, *aox1a* and *aox1d* mutants display increased sensitivity to salinity recovery, linking the capacity for “safe” proline breakdown to stress resilience [108]. This dual nature of proline—protective when it accumulates but potentially harmful when degraded too rapidly—explains why simply engineering higher proline content has not always improved crop performance.

Glycine betaine is synthesized from choline via a two-step oxidation, with betaine aldehyde dehydrogenase (BADH) as the key enzyme. Unlike proline, glycine betaine accumulation is restricted to certain families. Many important crops, including *Arabidopsis*, tomato, and potato, do not naturally accumulate glycine betaine to significant levels. Nevertheless, heterologous expression of *BADH* genes from stress-tolerant species in *Arabidopsis* or rice enhances tolerance to salt and drought, associated with reduced ROS accumulation, higher antioxidant enzyme activities, and improved photosynthetic performance [109,110]. Paradoxically, this successful engineering demonstrates that the pathway can be introduced without obvious negative effects, yet it has not been widely deployed in commercial crops. The likely reason is that the benefits are context-dependent: under mild stress, gains are modest, and under severe stress, the engineered pathway may not be sufficiently induced. A more fundamental question is why plants lost the capacity for glycine betaine synthesis in the first place. The answer may lie in the metabolic cost of diverting choline from membrane phospholipid synthesis, a trade-off not yet systematically investigated.

#### 2.4.2. Metal Chelators: Alleviating Heavy Metal Toxicity

Under heavy metal stress, plants activate mechanisms to sequester toxic ions. Here again, amino acids play central roles, both directly as metal-binding ligands and indirectly as precursors of metal-chelating peptides.

Histidine is unusual among the proteinogenic amino acids in having an imidazole side chain that coordinates transition metals with high affinity. In the nickel hyperaccumulator *Alyssum lesbiacum*, histidine concentrations increase dramatically under nickel stress, forming stable complexes with Ni^2+^ (Figure 3) [111]. In *Arabidopsis*, mutation of the histidine biosynthetic gene *ATP-PRT1* leads to nickel hypersensitivity, and exogenous histidine rescues this phenotype, indicating that even in non-accumulators, endogenous histidine synthesis contributes to basal nickel tolerance [112]. More recent work has shown that Ni–histidine complexes can generate ROS-Ca^2+^ signaling events, suggesting histidine not only chelates nickel but also modulates the plant’s stress signaling response [113,114]. This dual function has received surprisingly little attention.

The most important metal chelators in plants, however, are not free amino acids but phytochelatins (PCs). These are small, cysteine-rich peptides synthesized from glutathione by phytochelatin synthase (PCS). PCs bind cadmium, arsenic, mercury, and other toxic metals via their thiol groups, and the resulting complexes are transported into the vacuole (Figure 3). The *Arabidopsis cad1* mutant, which lacks functional PCS1, is extremely sensitive to cadmium and arsenic [115]. More recent work has revealed that PCS enzymes have additional functions beyond metal detoxification; for example, the Caenorhabditis elegans PCS can complement the metal sensitivity of the *Arabidopsis cad1* mutant but cannot fully restore its non-host pathogen resistance, suggesting PCS proteins have evolved distinct functions in different lineages [116]. In rice, two PCS genes (*OsPCS1* and *OsPCS2*) make different contributions to cadmium versus arsenic tolerance, and alternatively spliced transcripts of *OsPCS2* add another layer of regulatory complexity [117,118]. The implication is that phytochelatin synthesis is not a simple, conserved pathway but a system that has adapted independently to different metal challenges and plant lineages.

#### 2.4.3. Transcriptional Regulation of Amino Acid Metabolism and Signal Integration

The metabolic reprogramming of amino acids under abiotic stress is actively orchestrated by transcription factors that integrate stress signals with metabolic status. Among the most important are the bZIP transcription factors, particularly members of the group S1 subfamily (bZIP1, bZIP11, bZIP53). These factors directly bind to the promoters of amino acid metabolism-related genes—including *ASN1* and *ProDH*—thereby linking nutrient status with stress responses (Figure 3) [119]. Recent transcriptomic studies have revealed that bZIP1 and bZIP53 control a co-expression network governing amino acid catabolism, gluconeogenesis, and energy homeostasis during starvation and recovery [120].

Hormonal crosstalk adds another layer of regulation. ABA strongly induces *P5CS1* expression, while auxin and cytokinin may act antagonistically. The nitrate transporter NRT1.1 has been shown to regulate auxin transport, coupling nitrogen status to root system architecture [121]. What remains unknown is how these multiple, sometimes opposing, inputs are integrated at the promoter level. Are the same bZIP factors responsible for basal amino acid metabolism under unstressed conditions as for stress-induced reprogramming? Comparative studies across species are almost entirely lacking, despite the obvious agricultural importance.

What emerges from surveying the roles of amino acids in abiotic stress is a picture of metabolic flexibility constrained by context. Proline and glycine betaine are not always protective; their effects depend on stress severity, duration, and genetic background. Glutathione is not simply an antioxidant; it is a signaling molecule whose redox state influences gene expression. Phytochelatins are not just for metal detoxification; they have acquired additional functions in different lineages. The challenge for future research is to move beyond cataloging which amino acid accumulates under which stress, toward understanding the regulatory logic that governs when, where, and how much of each amino acid is deployed—and at what cost to growth.

Thus, under abiotic stress, plants rapidly repurpose amino acids as osmoprotectants, antioxidants, and metal chelators, but the degree of protection is highly context-dependent and comes at a significant metabolic cost, highlighting the need to better understand the regulatory logic that balances stress survival with continued growth.

### 2.5. Roles of Amino Acids in Response to Biotic Stress

Plants are continuously threatened by a diverse array of pathogens and herbivores. Unlike animals, plants rely on cell-autonomous defenses and systemic signals [122]. For decades, research focused on signaling hormones such as salicylic acid (SA), jasmonic acid (JA), and ethylene. More recently, attention has turned to the metabolic underpinnings of defense—and amino acids have emerged as far more than passive nutrients [123]. They serve as direct antimicrobial agents, precursors of phytoalexins, substrates for volatile signals that recruit natural enemies of herbivores, and even as immune elicitors. Yet the same amino acids that power defense can also nourish invading pathogens, creating a fundamental tension [123]. Unraveling how plants balance this trade-off is one of the most pressing questions in plant–pathogen biology.

#### 2.5.1. Direct Defense: Non-Protein Amino Acids and Phytoalexins

Some of the most potent antimicrobial and anti-herbivory compounds are non-protein amino acids (NPAAs) [124]. Plants produce over five hundred such NPAAs, and many function as effective chemical defenses by mistiming herbivore or pathogen metabolism. The arginine analogue L-canavanine, found in many legumes, is the classic example [125]. When ingested by non-adapted insects, canavanine is misincorporated into nascent proteins in place of arginine, causing protein misfolding and proteotoxic stress (Figure 3) [124]. These NPAAs are not merely passive toxins; plants that accumulate canavanine have evolved a resistant protein synthesis machinery that discriminates against the analogue [126]. This elegant molecular arms race illustrates a general principle: plants deploy amino acid analogues as “Trojan horses” to exploit the fidelity of the translation machinery in their attackers.

In *Arabidopsis thaliana*, the tryptophan-derived phytoalexin camalexin represents the most thoroughly studied amino-acid-based direct defense. Upon pathogen attack, camalexin is rapidly synthesized from tryptophan via indole-3-acetaldoxime (IAOx), catalyzed by the cytochrome P450 enzymes CYP79B2 and CYP79B3 [127,128]. From this branch point, IAOx can be channeled into camalexin or into indole glucosinolates, depending on the input of MYB transcription factors MYB34, MYB51, and MYB122 [42]. The functional importance has been demonstrated: the pad3 mutant shows extreme susceptibility to Botrytis cinerea [129]; the *cyp79b2 cyp79b3* double mutant is similarly compromised in camalexin production and basal immunity [127]. Recent studies have further shown that the unfolded protein response activates bZIP28 and bZIP60 transcription factors, which in turn upregulate *WRKY33* and drive camalexin and IAA production [130]. This connection reveals that camalexin synthesis is embedded within broader cellular stress responses.

What remains unresolved is the quantitative contribution of camalexin versus other tryptophan-derived metabolites to overall resistance. A comprehensive mutational analysis of the indolic network in *Arabidopsis* infected with *Colletotrichum higginsianum* revealed that only camalexin—not indole glucosinolates nor the recently described phytoalexin 4-OH-ICN—played a significant role in limiting fungal colonization [128]. This finding challenges the assumption that all branches of the tryptophan pathway contribute equally to immunity.

#### 2.5.2. Indirect Defense: Volatile Compounds and Priming

Beyond direct antimicrobial activity, plants also deploy amino-acid-derived molecules as airborne signals to recruit natural enemies of herbivores and to prime neighboring tissues. Green leaf volatiles (GLVs) are produced within seconds to minutes of tissue damage, with branched-chain amino acids serving as important precursors for volatile esters [131]. Upon herbivory, plants emit GLVs that attract predatory and parasitic insects and induce defense gene expression in undamaged parts of the same plant or in neighbors [132]. Recent work demonstrated that the GLVs Z-3-hexen-1-ol (Z3-HOL) and Z-3-hexenyl acetate (Z3-HAC) trigger rapid changes in protein phosphorylation in tomato cell cultures within 5 min, affecting pattern recognition receptors, MAPK cascades, calcium signaling proteins, and transcriptional regulators [132]. These findings suggest GLVs act not merely as attractants but as bona fide signaling molecules.

β-Aminobutyric acid (BABA) represents a different class of defensive molecule: a priming agent. BABA does not directly activate defense responses but “trains” the plant to respond more rapidly and robustly when subsequently challenged [133]. BABA primes defense reactions controlled by both SA-dependent and SA-independent pathways [134]. The molecular mechanism was elucidated when the aspartyl-tRNA synthetase IBI1 was discovered to function as the BABA receptor in *Arabidopsis* [135]. Binding of R-BABA to IBI1 primes a non-canonical defense activity of the synthetase, leading to the priming of multiple immune outputs. The *ibi1* point mutant completely loses BABA-induced resistance, providing definitive proof of IBI1’s receptor function [135]. Despite this mechanistic understanding, a significant translational gap remains: the phytotoxicity of BABA at effective doses has limited its agricultural application.

#### 2.5.3. The Dual Role of Glutamine in Immunity

Glutamine occupies a uniquely complex position at the intersection of nitrogen metabolism and plant immunity. As the primary amino acid product of the GS/GOGAT cycle, glutamine is both a central nitrogen carrier and a key regulator of cellular redox and energy status. Recent work has revealed that glutamine also functions as an immune signal [136]. *Arabidopsis* seedlings grown with glutamine as the sole nitrogen source upregulate stress- and defense-responsive genes, including *PR1*, *SARD1*, *WRKY54*, and *WAK1* (Figure 3). Exogenous glutamine enhances disease resistance against Pseudomonas syringae [136]. Notably, this induction is compromised in SA biosynthetic and signaling mutants, suggesting glutamine’s immune effects are at least partially mediated through the SA pathway [136].

However, the relationship between glutamine and immunity is not straightforward: the same molecule that primes defense also serves as a rich nitrogen source that pathogens can exploit. The amino acid transporter LHT1 plays a critical role in this duality. In the *Arabidopsis lht1* mutant, impaired glutamine uptake leads to constitutive activation of SA-dependent defense and broad-spectrum disease resistance—but also to reduced plant growth [137]. This suggests that it is not the absolute level of glutamine but its compartmentalized distribution and the resulting metabolic imbalance that determines whether the plant mounts a defense response.

What emerges from surveying the roles of amino acids in biotic stress is a picture of functional duality and metabolic tension. Non-protein amino acids protect by masquerading as harmless nutrients; volatile amino acid derivatives communicate danger across distances; glutamine simultaneously supports growth and triggers defense, while also creating a nutrient resource that pathogens can exploit. The same amino acid transporter that supplies nitrogen for protein synthesis can, when perturbed, activate full-blown immunity at the cost of stunted growth. This trade-off between defense and growth—mediated by amino acid metabolism and transport—is the central unresolved question in the field. Answering it will require a deeper understanding of how plants sense amino acid imbalances and the ability to experimentally separate the nutritional from the signaling functions of specific amino acids [123].

In conclusion, amino acids contribute to biotic stress responses through diverse mechanisms—ranging from direct antimicrobial action and volatile signaling to priming and immune modulation—yet the same molecules can also nourish pathogens, creating an inherent duality that forces plants to tightly regulate amino acid partitioning, a trade-off that sits at the heart of the growth-defense balance.

## 3. Conclusions and Perspectives

### 3.1. Summary of Key Conclusions

This review has traced our understanding of amino acids in plant biology—from obligate protein building blocks to multidimensional regulatory hubs that integrate carbon-nitrogen metabolism, signal transduction, stress adaptation, and developmental coordination. Several overarching themes have emerged. First, plants do not passively accumulate amino acids; they control their synthesis, allocation, and storage through multi-layered networks that include feedback inhibition, post-translational modification, and tissue-specific transport. The recent discovery of widespread substrate promiscuity among plant aminotransferases reveals that these networks possess a built-in redundancy and resilience that linear pathway models cannot capture [22]. Second, the signaling functions of amino acids—through GLR channels, the PII protein, and the TOR pathway—operate on vastly different timescales yet converge on the same problem: how to match growth and development with nutritional status. Third, stress responses depend crucially on the rapid repurposing of amino acid metabolism. Proline, glycine betaine, glutathione, and phytochelatins each illustrate a different facet of how plants use amino acids to protect themselves, but they also reveal the metabolic costs and trade-offs that come with such repurposing. Recognizing these trade-offs directly affects how we interpret the success or failure of engineering interventions aimed at improving stress tolerance.

### 3.2. Current Challenges and Key Scientific Questions

Despite substantial progress, fundamental gaps remain. Perhaps the most significant is the species bias of current knowledge. The overwhelming majority of mechanistic studies have been conducted in *Arabidopsis thaliana*. As we have noted, generalizations from *Arabidopsis* to cereals, legumes, and horticultural species frequently fail—whether in the absence of camalexin in rice, differences in betaine accumulation, or the species-specific regulation of TOR signaling [52]. Systematic comparative studies across major crop families are urgently needed.

A second major challenge is spatial and temporal heterogeneity. Conventional omics approaches average signals across entire organs, masking the cell-type-specific patterns on which developmental and stress responses depend. Single-cell transcriptomics has revealed striking heterogeneity in nitrogen responses across root cell layers [138]; similar approaches applied to metabolomics and signaling readouts will be essential.

Third, integration across scales—from molecular mechanisms to whole-plant physiology to ecosystem function—is largely missing. We know that root exudates contain amino acids that shape the rhizosphere microbiome [139], but we do not know how plants regulate the composition and quantity of these exudates, nor how microbial perception of amino acids feeds back onto plant physiology. Addressing these ecosystem-scale questions will require interdisciplinary collaboration.

### 3.3. Future Research Directions

#### 3.3.1. Mechanistic and Integrative Approaches

Technological innovations offer unprecedented opportunities to address the challenges identified above. Spatial multi-omics—combining single-cell transcriptomics, spatially resolved metabolomics, and proteomics—can construct cell-resolution “amino acid metabolic atlases” for model and crop species [140]. The power of such atlases lies in the hypotheses they generate. Answering these questions will require the development of genetically encoded biosensors for specific amino acids [141]. Real-time monitoring of subcellular amino acid dynamics would transform the field from static snapshots to dynamic understanding.

On the structural front, cryo-electron microscopy has yielded the first view of a GLR ligand-binding domain [142]; full structures of intact GLR tetramers, transporter complexes, and TOR signaling assemblies will reveal the molecular basis of signal recognition. However, structure alone is insufficient. We need to understand how these molecular machines operate in living cells.

A critical and often neglected direction is the development of predictive computational models of amino acid metabolic networks. The substrate promiscuity of aminotransferases makes metabolic network modeling particularly challenging but also essential. Current models are mostly static; they cannot predict how flux re-routes when a single enzyme is perturbed or when the plant experiences combined stresses. Building and validating such models will require kinetic data, but the effort is justified because only predictive models can guide rational engineering.

#### 3.3.2. Precision Design for Agricultural Applications

**Figure 4:**
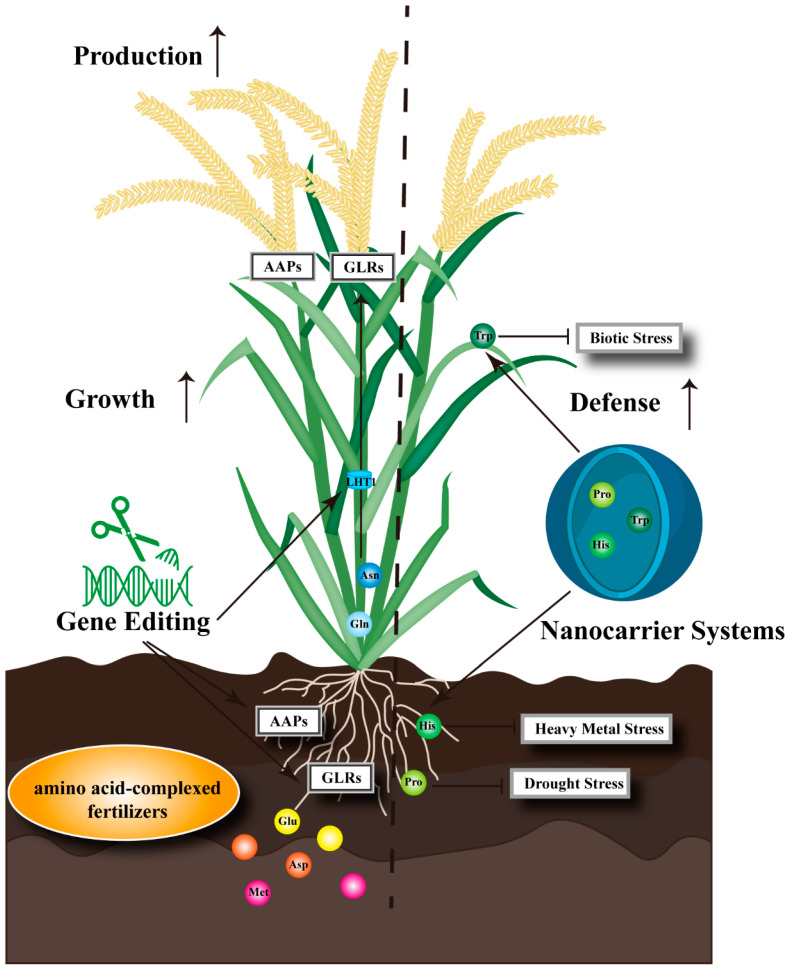
**Translating amino acid research into sustainable agricultural practices.** This diagram outlines prospective applications based on the advanced understanding of amino acid functions in plants. **Precision Biostimulants:** Development of tailored amino acid formulations (e.g., Proline for drought stress, Glycine Betaine for salinity, specific blends for nitrogen use efficiency) for foliar or soil application to enhance crop resilience and yield. **Precision Breeding & Genetic Engineering:** Utilization of gene editing tools (e.g., CRISPR/Cas) to modulate key nodes in amino acid metabolism, such as creating feedback-insensitive biosynthetic enzymes or optimizing amino acid transporter (e.g., AAP family) activity, aiming to improve seed nutritional quality, stress tolerance, and nitrogen allocation. **Smart Delivery Systems:** Employment of nano-carrier platforms (e.g., chitosan nanoparticles, lipid vesicles) for the targeted and controlled release of amino acids or their complexes to specific plant tissues, enhancing uptake efficiency and protection against degradation. These integrated strategies contribute to the goals of sustainable agriculture by reducing fertilizer input and enhancing crop performance under challenging environments. LHT1, lysine histidine transporter 1; Asn, asparagine; Gln, glutamine; His, histidine; Pro, proline; Trp, tryptophan; Glu, glutamate; Asp, aspartic acid; Met, methionine; AAPs, amnio acid permeases; GLRs, glutamate receptor-like channels.

The translation of basic knowledge into agricultural practice is underway, though at an early stage. The global biostimulants market is growing rapidly, and amino-acid-based products constitute a substantial segment. Among commercially available formulations, Terra-Sorb^®^ is an enzymatic hydrolysate containing free L-α-amino acids and micronutrients, commercialized in more than 50 countries. Another example is HICURE^®^, a highly concentrated biostimulant containing 19 amino acids. Field studies on these and related products have demonstrated yield improvements under drought and salinity in crops such as creeping bentgrass, rice, durum wheat, and soybean (Figure 4) [143,144,145,146].

Despite these successes, major challenges remain. Production costs are high, and limited economies of scale keep prices elevated. Lack of standardization across products complicates regulatory approval and farmer decision-making. Inconsistent field efficacy suggests that amino acid formulations work best when tailored to specific crops, stress types, and phenological stages, a level of precision that current off-the-shelf products rarely achieve. Future efforts should focus on cost-effective production, smart delivery systems (encapsulation, nano-carriers) that protect amino acids from degradation (Figure 4) [147], and the development of crop-specific formulations based on mechanistic understanding rather than empiricism.

#### 3.3.3. From Arabidopsis to the Field and Beyond

We return to the theme that has run throughout this review: the need to move beyond *Arabidopsis*. The tools of modern plant science—CRISPR gene editing, synthetic biology, high-throughput phenotyping—are now available for crop species (Figure 4). Precision breeding targeting amino acid transporters has already shown promise: in pea, simultaneous overexpression of AAP1 and a seed-specific amino acid transporter increased seed protein content without reducing biomass (Figure 4) [148]. This proof-of-concept should be extended to other crops and transporter families. Similarly, engineering feedback-insensitive versions of key biosynthetic enzymes has been attempted but rarely reached commercialization due to unintended metabolic consequences. The substrate promiscuity of aminotransferases [22] may explain some of these unintended effects: altering flux through one pathway inevitably affects others. This is a reason to adopt systems-level approaches that predict secondary effects before they occur.

Beyond the plant itself, ecosystem-level integration represents the frontier. Root exudates shape the rhizosphere microbiome [139]. We do not yet understand the rules of this chemical dialogue. Synthetic microbial communities (SynComs) and gnotobiotic systems are beginning to provide answers, but these approaches have been applied to only a few crop species.

Finally, we must confront the reproducibility crisis. Many of the studies cited in this review have been conducted under highly controlled laboratory conditions that bear little resemblance to field environments. Translating laboratory findings to the field requires rigorous validation under realistic conditions. Funding agencies and journals should encourage multi-environment validation for claims with agricultural relevance.

The challenges are substantial, but so is the payoff. A deeper understanding of amino acid multifunctionality will not only illuminate fundamental plant biology but also provide practical solutions for sustainable agriculture in an era of climate change. The transition from *Arabidopsis* to crops, from descriptive to predictive models, and from laboratory to field is not optional; it is the only path forward.

## Data Availability

This review article does not contain any new experimental data. All data analyzed during this study are included in the published articles cited in the reference list. Literature search strategy: A systematic literature search was conducted using PubMed, Web of Science, and Google Scholar for articles published up to March 2026. Search terms included combinations of ‘amino acids’, ‘plants’, ‘signaling’, ‘stress tolerance’, ‘nitrogen metabolism’, ‘biosynthesis’, and ‘transport’. Only peer-reviewed articles, reviews, and book chapters written in English were considered. References cited in retrieved articles were also screened for relevance. No publication date restrictions were applied for landmark discoveries, but priority was given to studies published in the last decade.
